# A Study Investigating the Value of Using a Novel Mobile Application as an Adjunct to the Traditional Method of Diagnosing Periodontal Diseases

**DOI:** 10.1002/cre2.70178

**Published:** 2025-07-24

**Authors:** Brian Maloney, Bahman Honari, Ioannis Polyzois

**Affiliations:** ^1^ Department of Restorative Dentistry and Periodontology Dublin Dental University Hospital, University of Dublin, Trinity College Dublin Ireland; ^2^ Department of Biostatistics Dublin Dental University Hospital, University of Dublin, Trinity College Dublin Ireland

**Keywords:** diagnosis, mobile application, periodontitis

## Abstract

**Background:**

Periodontal disease is a prevalent condition in the general population. An updated classification system in 2018 introduced major changes to how this disease is classified and has implications for management. Research has demonstrated challenges in reaching periodontal diagnoses with this new system, prompting the need for the development of resources to assist clinicians.

**Objectives:**

This novel research aims to determine the level of accuracy and reliability in the assignment of case definitions of periodontal diseases according to the 2018 classification using a specialized mobile application.

**Materials and Methods:**

Newly qualified dentists were recruited and assigned five random cases to classify according to the 2018 classification system. The collected data were analyzed to determine intra‐ and inter‐examiner accuracy and consistency.

**Results:**

The overall accuracy of staging was 84%. The correct grade was assigned in 96% of cases. The extent was accurate in 97%. Localized disease was more reliably diagnosed than generalized forms of the condition. When accounting for stage, grade, and extent, examiners demonstrated 76% accuracy. Inter‐examiner agreement was 62.5%.

**Conclusions:**

There was a high level of diagnostic accuracy and consistency in periodontal disease diagnosis when diagnostic software was used as an adjunct to assigning case definitions. Dedicated software like “PerioBrain” has the potential to improve diagnostic accuracy. Further research is warranted to investigate the use of this application in a clinical setting and for didactic teaching of a student cohort.

## 1 Introduction

The World Workshop on the Classification of Periodontal and Peri‐implant Diseases and Conditions was convened in 2017 under the auspices of the European Federation of Periodontology (EFP) and the American Academy of Periodontology (AAP) (Caton et al. 2018). This collaborative effort of multiple expert working groups is proposed to formulate a new system for the classification of periodontal conditions to align with emerging scientific evidence gathered from the ensuing decades. This resultant framework introduced major changes in disease descriptors and terminology for periodontal health and disease, including the concept of disease staging and grading.

The integration of novel classification systems into clinical practice and research requires time and training to overcome the associated learning curve. Despite considerable dissemination efforts, the literature suggests inadequacies in the consistency and accuracy of assigning periodontal case definitions using this system (Marini et al. 2021; Kakar et al. 2022).

A correct diagnosis is essential to dictate appropriate, evidence‐based treatment decisions (Herrera et al. 2022). The inability of clinicians to consistently establish a correct diagnosis may lead to inappropriate therapies, ineffective communication with patients and members of the dental team, and delayed referral to secondary treatment centers (Jayawardena et al. 2021), potentially compromising prognosis.

Notwithstanding these challenges, evidence has demonstrated improvements in the consistency of assigning correct diagnoses following training and experience in the new classification of periodontal and peri‐implant diseases (Ravidà et al. 2021). While the EFP and associated national societies have introduced algorithms (Tonetti and Sanz 2019) and flowcharts (Sutthiboonyapan et al. 2020) to aid clinicians, the need for the introduction of complementary diagnostic software tools has also been recommended (Marini et al. 2021; Tonetti and Sanz 2019). Such software tools and applications use inputted patient data to generate decisions regarding diagnosis and treatment planning. While such systems have been used in healthcare, research on their use and effectiveness in dentistry is limited (Mendonça 2004).

“PerioBrain” is a user‐friendly digital periodontal diagnostic tool developed in English to assist dental healthcare professionals and students in establishing and verifying the classification of periodontal and peri‐implant diseases. The current study aims to ascertain the accuracy and reliability in the assignment of case definitions of periodontal diseases according to the 2018 classification when a specialized mobile application is used to assist clinicians in formulating a diagnosis.

## 2 Materials and Methods

### 2.1 Study Design

Study participants included ten newly qualified dental practitioners who were required to formulate periodontal diagnoses according to the 2018 Classification system on five randomly selected cases from a total pool of 10. Baseline clinical and radiographic as well as anamnestic information was provided. The responses were analyzed to determine the diagnostic accuracy and the inter‐rater agreement.

Participants were blinded to one another and independently examined each of the provided cases. The only tool available to the examiners was the “PerioBrain” application. Participants were required to provide a periodontal diagnosis for each case based on the provided anamnestic information according to the 2018 classification system. Once each participant completed the five cases, the data collection forms were returned for statistical analysis. The study was completed in a specified area within the School of Dental Science on a once‐off occasion. The study was conducted according to the Guidelines for Reporting Reliability and Agreement Studies (GRRAS) (Kottner et al. 2011).

### 2.2 Ethical Approval

An application for ethical approval was submitted to the Dental School Research Ethics Committee in May 2024. Reference REAMs No: 3082. Anonymous baseline clinical and radiographic documentation of periodontitis cases from subjects attending periodontology referral clinics in the Dublin Dental University Hospital between January and December 2023 was collected. Subjects provided informed consent for the use of their anonymous data for research and educational purposes.

### 2.3 “PerioBrain” Technical Specifications

“PerioBrain” is a free mobile application developed by SHO Me Digital Ltd. The app can be used on devices running Android (version 4.1 and above) or iOS (version 8 and above). The decision tree algorithm is composed of several questions regarding specific patient parameters to identify, classify, and grade periodontal diseases. There are three main sections: patient history (age, smoking status, presence of diabetes), clinical examination (presence of clinical attachment loss, pocketing, bleeding on probing, mobility, bony defects, furcation, tooth loss due to disease, bite collapse, drifting of teeth), and radiographic features (bone loss, vertical bony defects). This information is inputted by the clinician, and a periodontal diagnosis based on the class is generated.

**Figure 1:**
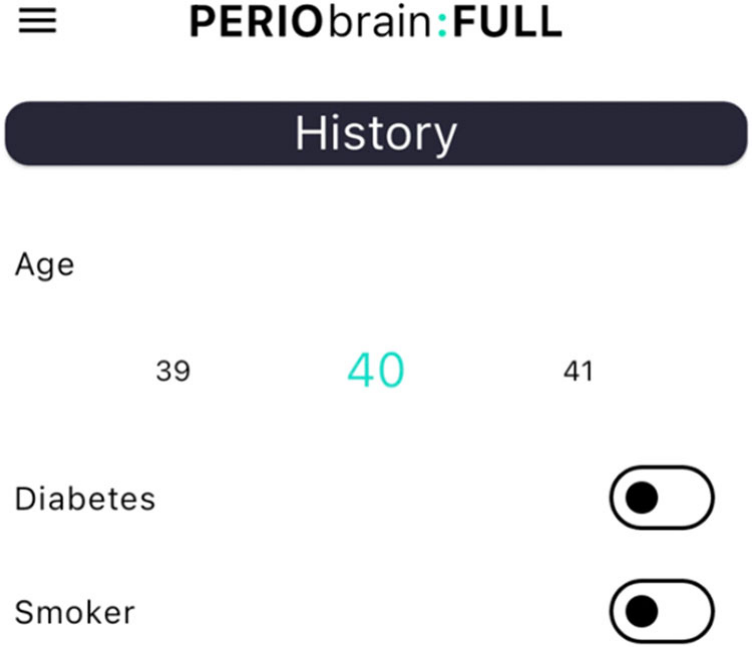
Clinical history.

**Figure 2:**
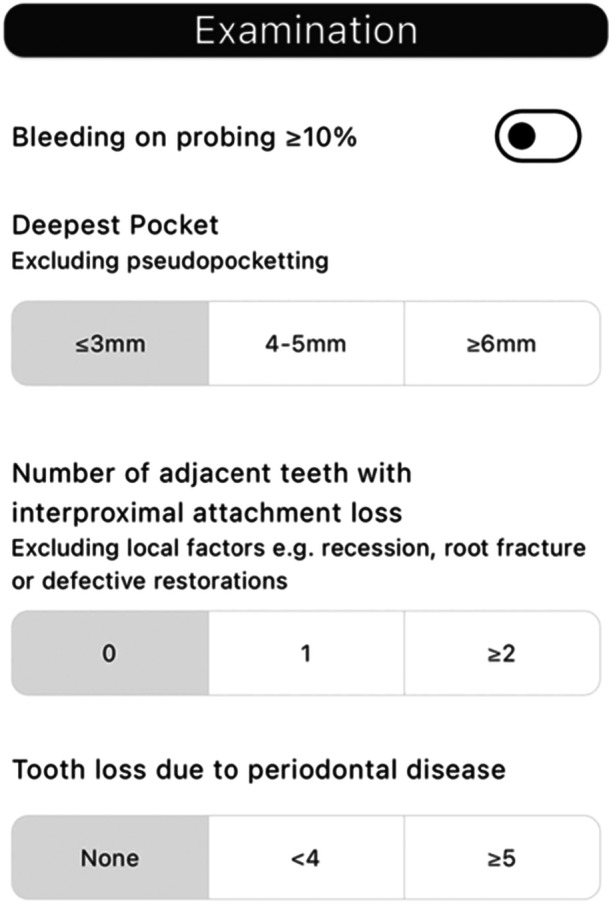
Clinical periodontal parameters.

**Figure 3:**
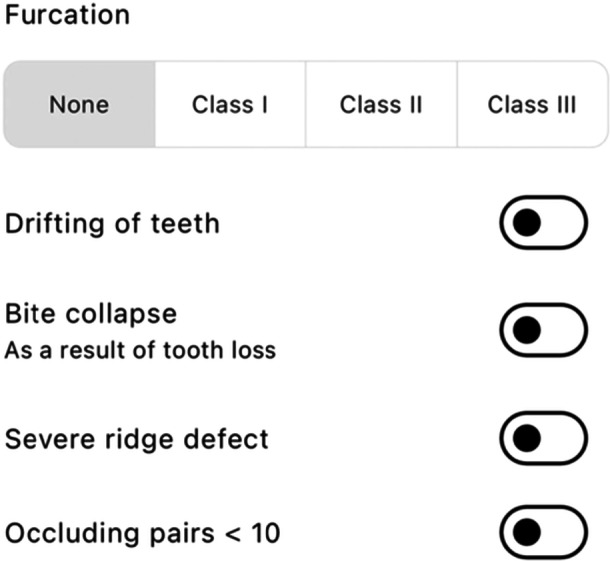
Clinical periodontal parameters.

**Figure 4:**
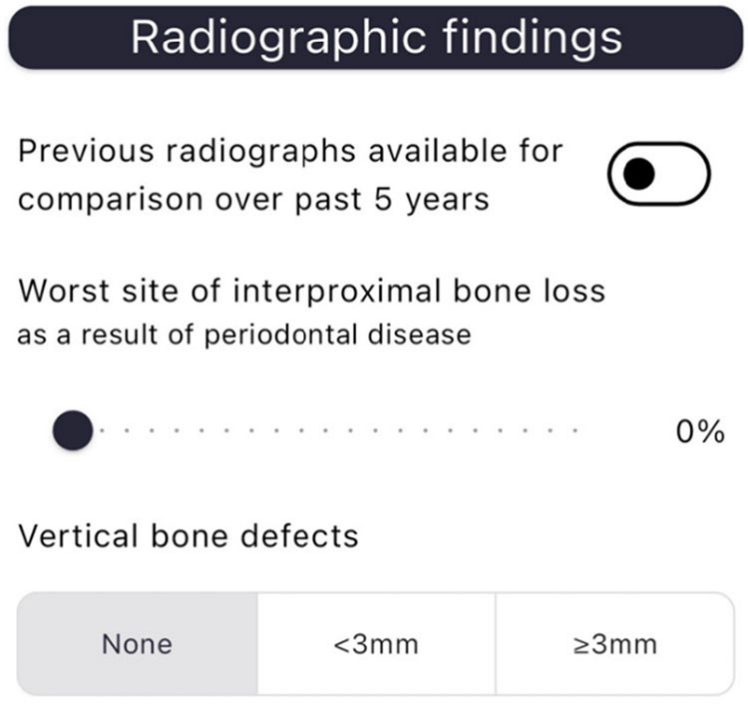
Radiographic parameters.

**Figure 5:**
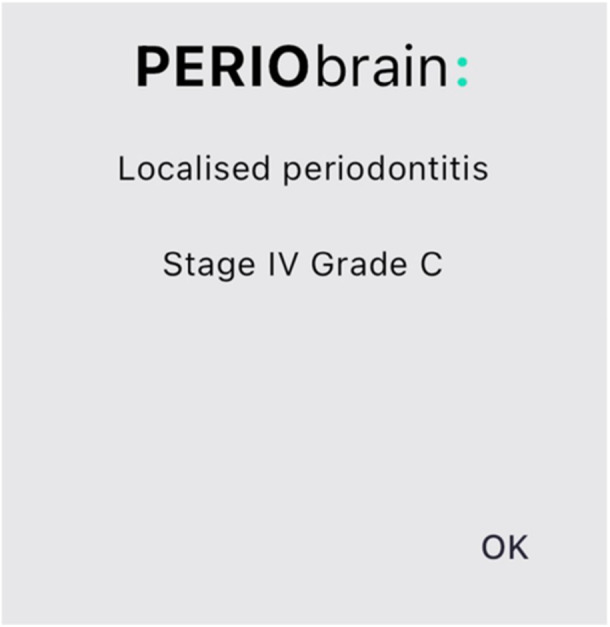
Generation of stage and grade for periodontal disease based on the parameters discussed earlier.

The application is formatted into three primary sections: “PerioBrain” simple classification is based on the British Society of Periodontology (BSP) screening tool for periodontal diseases. “PerioBrain” Full (complete international classification) and “Peri Implant Brain” (complete international classification). Figures 1, 2, 3, 4, 5 demonstrate how a diagnosis is developed using the application.

### 2.4 Training of Examiners

All study participants were newly qualified general dental practitioners. A PowerPoint presentation of the application and its functions and uses was delivered, and participants were given two mock cases to trial the functionality of the app. This initial meeting resulted in the identification of errors in the software, which were subsequently updated before the study was carried out.

### 2.5 Gold‐Standard Diagnosis

Photographs, clinical, and radiographic data of 10 periodontal cases were used for this study. One of these cases can be seen in Figure 2. Two experienced periodontists were designated to assign the correct diagnosis to all cases. This final diagnosis was confirmed by a third senior academic‐periodontist.

### 2.6 Selection and Preparation of Cases

Participants were provided with a document containing five randomly selected cases (out of the 10 originally prepared). The allocation of cases was determined using a generic online random number generator. The information provided included the patient's history (medical, social, and dental history), clinical photos, full periodontal charting, and radiographs. The full periodontal chart provided information on the periodontal probing depths at 6 sites per tooth, recession at 6 sites per tooth, clinical attachment loss at 6 sites per tooth, and, if present, degree of mobility and furcation involvement. Radiographs provided included, at a minimum, an orthopantomogram taken at the time of clinical examination. If peri‐apical radiographs or older orthopantomograms were available, they were included and dated. Based on the findings from a pilot study conducted by the authors, a decision was made to focus exclusively on cases at the severe end of the spectrum, specifically Stages III and IV. The pilot study revealed that there were no significant challenges in identifying cases with mild periodontal disease or in distinguishing between Stages I and II, or between Stages II and III. Including these milder cases might have created a false impression of greater accuracy and reliability of the mobile application.

### 2.7 Outcomes

The primary outcome for the study was the accuracy of diagnosis reached by the examiners, across stage, grade, and extent assigned to each of the provided cases. Secondary outcomes included overall inter‐examiner reliability in diagnosing periodontal and peri‐implant disease when using the “PerioBrain” application as an adjunct.

### 2.8 Sample Size

A convenience sample was used to select the examiners for this study, selected from a group of 13 newly qualified dental practitioners working in a single institution. All eligible participants were invited to take part, and participation was voluntary. Recruitment beyond this group was not feasible due to institutional and practical constraints. The relatively high rate of participation of 71% is sufficient for clinician‐based research and supports the adequacy of the sample. This is mirrored by previous reliability studies. (Marini et al. 2021; Cairo et al. 2010; Isaia et al. 2018; Rotundo et al. 2015). A convenience sample, therefore, allowed timely and efficient data collection within the available workforce with sufficient statistical power to detect clinically meaningful differences in diagnostic accuracy.

### 2.9 Statistical Analysis

Descriptive statistics were employed for agreement between the examiners' diagnoses and the gold standard. Inter‐examiner reliability was assessed via the weighted mean of the agreement percentage for the pairs of examiners in the study.

## 3 Results

### 3.1 Characteristics of Cases

The characteristics of the 10 included cases can be seen in Table 1.

**Table 1:** Characteristics of the provided 10 cases.

	Frequency	Percentage
Characteristics		
Male	3	30%
Female	7	70%
Smoking status		
> 10	2	20%
< 10	1	10%
Never	7	70%
Diabetes (HbA1C)		
> 7%	0	0%
< 7%	1	10%
No	9	90%
Age		
20–29	2	20%
30–39	1	10%
40–49	6	60%
50–59	1	10%
Stage		
I	0	0%
II	0	0%
III	4	40%
IV	6	60%
Grade		
A	0	0%
B	0	0%
C	10	100%
Extent		
Localized	1	10%
Generalized	9	90%

### 3.2 Agreement With Gold Standard

**Figure 6:**
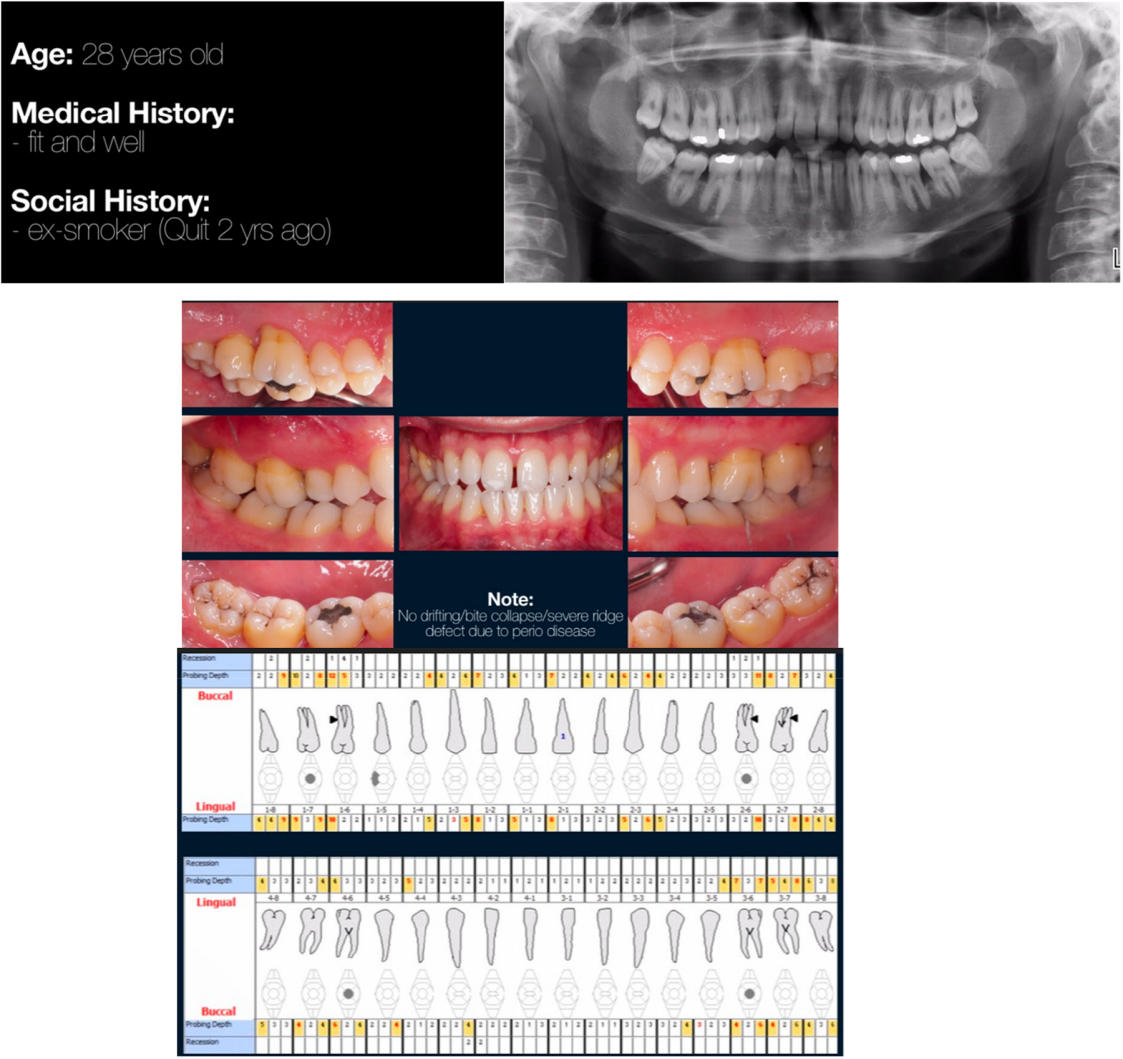
Example of case presentation and details provided to examiners.

The accuracy for the staging of cases was 84%. In relation to cases with Stage III disease, the accuracy was 100%. This number was reduced to 73% for cases of Stage IV disease (Table 2 and Figure 6).

**Table 2:** Accuracy of diagnosis of provided cases by stage, grade, extent, and overall diagnosis.

	Cases (*n*)	% Correct diagnosis
Stage		
III	4	100%
IV	6	73%
Grade		
C	10	96%
Extent		
Localized	1	100%
Generalized	9	97%
Overall diagnosis		
Stage/Grade/Extent		76%
One point difference		
2 out of 3		92%

**Figure 7:**
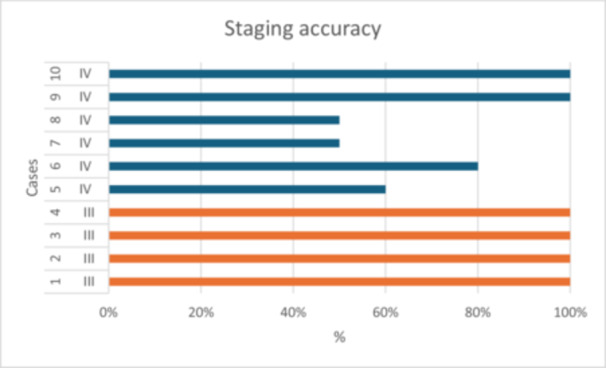
Accuracy of staging of cases.

The correct grade was assigned by examiners for 96% of cases. Where the incorrect grade was given, examiners tended to underestimate disease (Table 2 and Figure 7).

**Figure 8:**
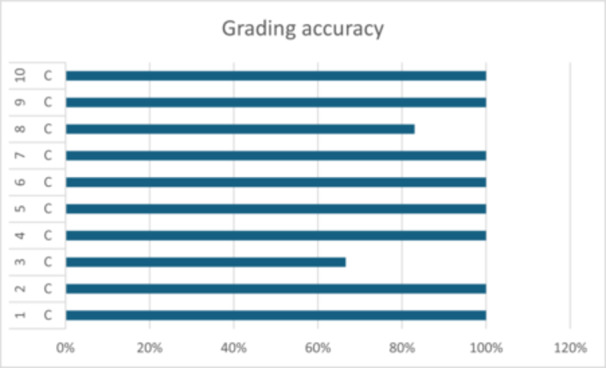
Accuracy of grading of cases.

The combined accuracy of disease extent for all cases in the study was calculated at 97%. Examiners demonstrated 100% accuracy when identifying localized disease. The correct identification of generalized disease amounted to 96% (Table 2 and Figure 8).

**Figure 9:**
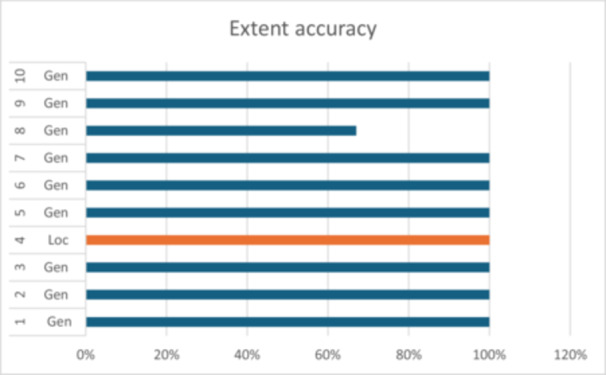
Accuracy of extent identification of cases.

Complete agreement for the overall periodontal diagnosis, accounting for stage, grade, and extent, was observed in 76% of cases. A total of 92% of cases were correctly identified within a one‐point difference of the true diagnosis (Table 2 and Figure 9).

Accuracy of diagnosis per case for stage, grade, and extent can be seen in Table 3.

**Table 3:** Accuracy of diagnosis per case for stage, grade, and extent.

	Stage			Grade			Extent		
Case	Gold standard	Agreement	Incorrect dx	Gold standard	Agreement	Incorrect dx	Gold standard	Agreement	Incorrect dx
1	III	100%		C	100%		Gen	100%	
2	III	100%		C	100%		Gen	100%	
3	III	100%		C	67%	B	Gen	100%	
4	III	100%		C	100%		Loc	100%	
5	IV	60%	III	C	100%		Gen	100%	
6	IV	80%	III	C	100%		Gen	100%	
7	IV	50%	III	C	100%		Gen	100%	
8	IV	50%	III	C	83%	B	Gen	67%	Loc
9	IV	100%		C	100%		Gen	100%	
10	IV	100%		C	100%		Gen	100%	
Accuracy									
	III	100%					Gen	96%	
	IV	73%					Loc	100%	
	Overall	**84%**			**95%**			**97%**	

### 3.3 Inter‐Examiner Agreement

Inter‐examiner agreement was determined by calculating the percentage of common correct diagnoses for each pair of examiners. The incorrect diagnosis of a case, which was agreed upon by paired examiners, was excluded. The total number of common cases between each pair of examiners was used as the weight of the agreement percentage. The overall agreement level was therefore calculated as the weighted mean of the agreement percentage for all pairs of examiners. The result showed the overall inter‐examiner agreement of 62.5% (Table 4).

**Table 4:** Weighted mean of the agreement percentage for all pairs of examiners.

Examiner 1	Examiner 2	Common cases	Correct agreement only	Agreement	Weight (*n*/5)	Weighted agreement
R1	R2	2	1	0.5	0.4	0.2
R1	R3	3	1	0.333	0.6	0.2
R1	R4	3	1	0.333	0.6	0.2
R1	R5	2	1	0.5	0.4	0.2
R1	R6	2	0	0	0.4	0
R1	R7	1	1	1	0.2	0.2
R1	R8	3	2	0.667	0.6	0.4
R1	R9	3	3	1	0.6	0.6
R1	R10	3	2	0.667	0.6	0.4
R2	R3	3	1	0.333	0.6	0.2
R2	R4	2	1	0.5	0.4	0.2
R2	R5	2	1	0.5	0.4	0.2
R2	R6	3	2	0.667	0.6	0.4
R2	R7	3	2	0.667	0.6	0.4
R2	R8	2	1	0.5	0.4	0.2
R2	R9	2	2	1	0.4	0.4
R2	R10	2	2	1	0.4	0.4
R3	R4	4	2	0.5	0.8	0.4
R3	R5	2	1	0.5	0.4	0.2
R3	R6	2	0	0	0.4	0
R3	R7	2	1	0.5	0.4	0.2
R3	R8	3	3	1	0.6	0.6
R3	R9	1	1	1	0.2	0.2
R3	R10	2	2	1	0.4	0.4
R4	R5	3	2	0.667	0.6	0.4
R4	R6	3	2	0.667	0.6	0.4
R4	R7	1	0	0	0.2	0
R4	R8	4	2	0.5	0.8	0.4
R4	R9	2	2	1	0.4	0.4
R4	R10	1	0	0	0.2	0
R5	R6	3	2	0.667	0.6	0.4
R5	R7	2	2	1	0.4	0.4
R5	R8	2	1	0.5	0.4	0.2
R5	R9	3	3	1	0.6	0.6
R5	R10	1	1	1	0.2	0.2
R6	R7	3	1	0.333	0.6	0.2
R6	R8	2	0	0	0.4	0
R6	R9	3	2	0.667	0.6	0.4
R6	R10	1	0	0	0.2	0
R7	R8	1	0	0	0.2	0
R7	R9	2	2	1	0.4	0.4
R7	R10	3	3	1	0.6	0.6
R8	R9	3	2	0.667	0.6	0.4
R8	R10	2	2	1	0.4	0.4
R9	R10	2	2	1	0.4	0.4
**Mean**						**0.625**

## 4 Discussion

**Figure 10:**
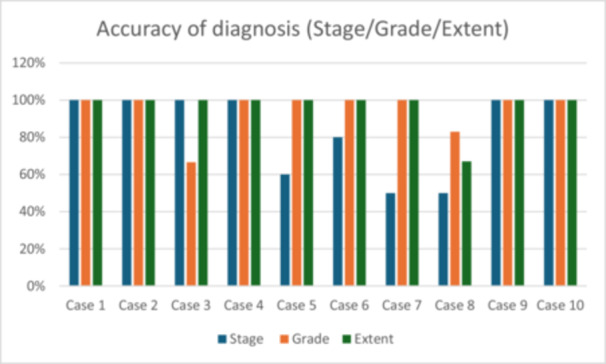
Accuracy of diagnosis of provided cases.

This study proposed to ascertain the value of a novel mobile application to aid general dental practitioners in the diagnosis of cases of periodontal disease according to the 2018 EEP/AAP Classification. The results demonstrate a high overall diagnostic accuracy for the provided cases. Concordance between examiners regarding complete agreement with the gold standard diagnosis for all three components was also high. The consistency of assigning the correct grade and extent to the provided cases was excellent, and, while lower, the consistency of staging of cases was also high (Figure 10).

The classification of periodontal disease has undergone several revisions and modifications over the last three decades (Ravidà et al. 2021). The most recent iteration in 2018 introduced a major revision of how we classify periodontal disease through a novel multidimensional staging and grading system. As new evidence emerges, the 2018 system is amenable to revision and modification to ensure periodontal care continues to be evidence‐based.

The implementation of a novel classification system in periodontology was likely to constitute a challenge. Marini et al. concluded that, while intra‐examiner reliability of assigning periodontal case definitions in a study cohort was favorable, inter‐examiner reliability was only moderate (Marini et al. 2021). Among those investigated, general dentists were found to have the lowest scores. This finding was mirrored by Jayawardena et al. on patient referrals to a periodontology department in the United Kingdom. Whilst some authors have found high inter‐examiner agreement (Ravidà et al. 2021; Abrahamian et al. 2022), the overall accuracy for all three diagnostic components (stage, grade, and extent) was low (47.2%) (Marini et al. 2021). This may be in part due to the novel nature of the system, differing nomenclature, or the extent of detailed information that needs to be considered and processed to formulate a diagnosis (Sutthiboonyapan et al. 2020). The existence of gray zones has been highlighted previously, which further complicates diagnosis (Sanz et al. 2020).

The inability to consistently establish a correct diagnosis may lead to ineffective communication with patients and among the dental team, an inability to understand and translate available scientific literature to everyday practice, and most importantly, a failure to translate individual patient findings to a precise treatment strategy that utilizes the recently published EFP S3‐ treatment guidelines (Sanz et al. 2020; Herrera et al. 2022). Therefore, the overall conclusion from these works is that there is a need for additional training and education for dental practitioners in the 2018 Periodontal and Peri‐Implant disease classification framework.

Education and training have been demonstrated to improve the accuracy of the application of the periodontal classification system (Ravidà et al. 2021). The literature suggests that when dental practitioners or students receive training and gain experience using the classification, they can be consistent in assigning periodontal diagnoses (Abrahamian et al. 2022). A novel approach to aid practitioners in improving the accuracy and consistency of periodontal diagnosis may be through the use of an easily accessible diagnostic software via a mobile application. A recent study highlighted the need for additional efforts in the field of periodontology to improve the training of general dentists and suggested the use of dedicated software for this purpose (Marini et al. 2021). Clinical decision‐support systems (CDSSs) are computer programs designed to assist health professionals in making clinical decisions based on expert support. It is believed that these modern tools could help achieve these goals in less time and with less effort compared to traditional methods.

Mobile applications that integrate CDSSs have been shown to have the potential to improve the accuracy and promptness of clinical diagnosis and management in healthcare (Machado et al. 2018). This medium can assist clinicians via a step‐by‐step input of the relevant parameters. Provided accurate data is input, this software will generate the correct periodontal diagnosis. Such systems have the potential to streamline the diagnostic and prognostic process, improve overall accuracy, and assist clinicians in selecting the most appropriate treatment strategies.

The applicability of CDSS in diagnosing periodontal disease according to the 2018 classification system has been recently investigated. Overall, results have been promising (Parsegian et al. 2024; Marini et al. 2024) with participants reporting an increase in confidence in diagnosing periodontal disease with the use of these applications (Parsegian et al. 2024).

In comparison to similar reliability studies, the current research demonstrates a high level of diagnostic accuracy for examiners when using the “PerioBrain” application to aid in diagnosis. To assess accuracy, each examiner's case definitions were compared with those provided by the gold standard examiner. Concordance in similar studies has been reported as moderate, in the region of 53% (Parsegian et al. 2024) and 54% (Marini et al. 2024).

Consistency of disease staging was 84%. This finding is mirrored in the work of Marini et al. wherein general dentists demonstrated 81% accuracy in disease staging (Marini et al. 2024). While all cases with Stage III disease were correctly identified by all examiners, there were some challenges in the identification of cases with Stage IV disease, especially Cases 7 and 8. This may be due to the failure of examiners to identify key clinical features that would escalate a case from Stages III to IV.

The accuracy of grading was higher than previously reported in the literature. Amongst the cases, 95% were graded correctly. This is in contrast to Marini et al. who demonstrated a 61% degree of concordance between the grade assigned and the gold standard diagnosis (Marini et al. 2024).

The extent of disease for periodontitis is classified as localized, generalized, and molar‐incisor pattern. The established method to calculate the extent is the percentage of teeth (not sites) at the stage‐defining severity level. In comparison to previous studies (Marini et al. 2024), participants in this study demonstrated a high level of accuracy in calculating the extent of periodontal disease present (97%).

Diagnostic errors have been highlighted as a primary cause for medico‐legal claims in healthcare (Berner and Graber 2008). Over or under‐estimation of disease has the potential to lead to an inappropriate treatment selection, delayed referral, and worse overall prognosis (Marlow et al. 2018). Incorrect diagnoses assigned to cases in this study were attributed to underestimation rather than overestimation. This is similar to previous research conducted in the UK, which found an underestimation of both stage and grade by practitioners referring cases of periodontal disease to a specialist center (Jayawardena et al. 2021). Approximately 40% of referrals underestimated the stage, and 39% underestimated the grade. This is in contrast to Marini et al. found that cases which were misdiagnosed were due to an overestimation of the degree of disease present (Marini et al. 2024).

On average, and taking into account the weight of each disagreement, the examiner pairs agreed on 62.5% of their ratings. This suggests moderate agreement, and it may point to areas where examiner calibration or consistency could be improved.

Case 8 demonstrated a challenge for examiners in this study. Examiners were more likely to give the incorrect diagnosis for stage (50%), grade (83%), and extent of disease (67%) compared to other cases. The case in question relates to a 48‐year‐old female with clinical attachment loss of 13 mm, mobility of her lower incisors, 60% bone loss with more than 30% of teeth affected with known Type II diabetes mellitus and an HbA1c > 7%. The gold standard diagnosis was generalized periodontitis, Stage IV, Grade C Participants tended to underdiagnose the case as Stage 3 Grade B. This disparity may be attributable to a failure to incorporate the patient's HbA1c into the grading of the case.

The current study has several limitations that should be considered when interpreting the results. Inclusion was limited to newly qualified general dental practitioners only. Recruitment of examiners was only conducted in a single institution. Regarding the included cases, there is a limited number in this study when compared to others (Marini et al. 2024; Parsegian et al. 2024). Several conditions included in the 2018 classification were not included, such as peri‐implant disease, gingivitis, and disease on a reduced periodontium. One limitation worth noting in particular relating to our study is the absence of a formal baseline knowledge assessment before using the application. However, all participants were newly qualified dentists with similar levels of recent clinical education and training. This homogeneity likely minimized differences in baseline diagnostic information between individuals. Future work should incorporate baseline assessments of participants and individuals with different levels of experience to explore how individual variations in knowledge/experience influence the diagnostic reliability of this application. In addition, while the current study, focusing solely on Stage III/IV periodontitis cases, offers a rigorous test of the app's validity, it is worth noting that including Stage I cases—deliberately excluded here—could have added value. Although Stage I cases can sometimes be misclassified as gingival health or reduced periodontium, they are generally much easier to diagnose. In a larger study, including a small proportion of such cases would likely have minimal impact. However, given our limited sample size, the inclusion of Stage I cases risked artificially inflating diagnostic performance. Overall, findings in the study herein should not be generalized to all periodontal diagnoses.

The strengths of this study include its demonstration of the use of a novel educational application to assist general dental practitioners in reaching diagnoses of cases of periodontal disease according to the 2018 classification framework. In comparison to previous research, this study investigates the accuracy of diagnosis in a cohort of general dental practitioners. This group is known to have difficulties with periodontal diagnosis when using the new periodontal framework (Marini et al. 2021) and inevitably sees a high proportion of patients in the primary care setting with periodontal disease. Tools like “PerioBrain” assist by organizing periodontal data and supporting clinicians in the classification process, but they still rely heavily on the practitioner's input and judgment. With the increasing integration of artificial intelligence in medical diagnostics, the future may lie in the development of AI‐assisted periodontal tools that seamlessly combine radiographic analysis with clinical periodontal data to deliver a comprehensive diagnosis. These advanced tools could also provide evidence‐based treatment recommendations, offer risk assessments based on the patient's overall health, and enable efficient data sharing between general dentists and specialist periodontists. This would ensure continuous monitoring and ultimately lead to improved patient outcomes. Until this vision is fully realized, applications like “PerioBrain” can still play a crucial role in helping general practitioners make accurate diagnoses.

## 5 Conclusion

Accurate and consistent classification of periodontal disease according to its severity, extent, risk of progression, and associated risk factors is fundamental to guiding clinical decision making and scientific discovery. The purpose of an accurate diagnosis is to support clinicians in prognostication and treatment planning for patients. The current research demonstrates a high level of diagnostic accuracy and consistency in periodontal disease diagnosis when diagnostic software is used as an adjunct to assigning case definitions. The use of dedicated software such as “PerioBrain” has the potential to improve diagnostic accuracy among dental practitioners. Future research is needed, however, to ascertain the benefit of such an application in the clinical setting, as well as its applicability for didactic teaching of a student cohort.

## Author Contributions

Brian Maloney contributed to the conception and design of the study, contributed to data collection, and critically revised the manuscript. Ioannis Polyzois contributed to the conception and design of the study and critically revised the manuscript. Bahman Honari performed the statistical analysis.

## Ethics Statement

Ethical approval was granted by the Dublin Dental School Research Ethics Committee in May 2024. Reference REAMs No: 3082.

## Conflicts of Interest

The authors declare no conflicts of interest.

## Data Availability

The data that support the findings of this study are available from the corresponding author upon reasonable request. Data is available upon request.
